# ISMB/ECCB Rebooted: 2015 Brings Major Update to the Meeting Program

**DOI:** 10.1371/journal.pcbi.1004320

**Published:** 2015-06-11

**Authors:** Christiana N. Fogg, Diane E. Kovats

**Affiliations:** 1 Freelance Science Writer, Kensington, Maryland, United States of America; 2 International Society for Computational Biology, La Jolla, California, United States of America

The 23rd Annual International Conference on Intelligent Systems for Molecular Biology and the 14th European Conference on Computational Biology (ISMB/ECCB 2015) is shaping up to be a phenomenal meeting, with world-renowned keynote speakers and changes in the meeting format that aim to make it a more streamlined and user-friendly conference. The joint ISMB/ECCB conference is held biennially and is the flagship meeting of the International Society for Computational Biology (ISCB). The meeting will take place at the Convention Center Dublin, Ireland, from July 10–14, 2015, and will bring together scientists from across the globe working in a broad range of computational biology—related disciplines, including genomics, structural biology, proteomics, data mining, machine learning, and systems biology. Online registration is open until June 26: https://www.iscb.org/ismbeccb2015-registration.

In the past, presentations at the meeting have been organized according to tracks, namely, the Proceedings, Highlights, and Late Breaking Research tracks. In 2015, all oral presentations will be presented in broad thematic areas. As in the past, submissions accepted into the highly selective Proceedings track will be published in a special ISMB/ECCB Proceedings issue of *Bioinformatics*.

Conference co-chairs Alex Bateman, Janet Kelso, and Des Higgins and the conference committee undertook the reorganization effort and came up with five thematic areas: Genes, Proteins, Systems, Disease, and Data. Batemen said, “The idea of themes is an obvious way to organize the talks. But, selecting a small number of themes that represented all computational biology was challenging. Of course, many talks will potentially fit across several themes. Time will tell whether these need any tweaking for future meetings.”

Kelso believes the new organization will benefit attendees and said, “We hope that organizing the meeting more thematically will mean that attendees have an easier time identifying sessions that are relevant and interesting to them.”

As in the past, the keynote speaker lineup features world-class scientists. The speakers include Amos Bairoch of the Swiss Institute of Bioinformatics (ISCB Fellows Keynote), Cyrus Chothia of the Medical Research Council (MRC) Laboratory of Molecular Biology (Senior Scientist Award winner), Eileen Furlong of the European Molecular Biology Laboratory, Curtis Huttenhower of the Harvard T. H. Chan School of Public Health (Overton Prize winner), 2013 Nobel Laureate Michael Levitt of Stanford University, and Kenneth Wolfe of University College Dublin.

The main conference program takes place from Sunday, July 12, through Tuesday, July 14. In the tradition of previous ISMB/ECCB meetings, special interest group and satellite meetings will occur prior to the conference on Thursday, July 10, and Friday, July 11. This year, the tutorials and workshops that usually precede the main meeting are being replaced by applied knowledge exchange sessions (AKES). The AKES are scheduled for Saturday, July 11, and are designed to provide interactive educational and knowledge exchange opportunities for attendees. AKES will also provide a chance for junior principle investigators to meet and exchange career advice. Six AKES have been scheduled:

AKES 01: Applied Knowledge Building Networks for Translation: How to Use DREAM Challenges and the Synapse Platform as a Research Strategy

AKES 02: Cytoscape 3 App Development: Variations on a Theme—“Hello World”

AKES 03: Bioinformatics Software Testing and Quality Assurance

AKES 04: Using Biological Cyberinfrastructure to Scale Science and People—Applications in Data Storage, HPC (High-Performance Computing), Cloud Analysis, and Bioinformatics Training

AKES 05: Open-Access, Cloud-Based, Individual-Level Clinical Trials Data—Sharing, Dissemination and Analyses

AKES 06: Junior Principal Investigator

ISMB/ECCB 2015 will also include special sessions, poster presentations in the exhibit hall, the Student Council Symposium, and social activities. This year’s meeting promises to be an eagerly anticipated reboot of ISMB/ECCB, with great science and collaboration at its core.

